# Medical Colleges and Journals

**Published:** 1861-06

**Authors:** 


**Medical Colleges and Journals.**

In these exciting times the stability of institutions of learning, particularly Medical Colleges, will be put to the test. Some, no doubt, will suspend temporarily, others permanently. This will be more particularly true of those in the old Union, which have heretofore obtained support from the now Confederate States of America. They cannot survive the change in government affairs. These institutions, located in the North, and having lived, flourished and prospered by Southern patronage, now droop, wither and die, from the attempt of their section to kill the goose, impatient for possession of all the golden eggs. While Southern colleges, so far from loosing patronage from a division of the country, will profit by students remaining at home, yet, in the excited state of the country, less interest will be felt in every branch of learning, (except that of military,) and, therefore, no very great prosperity need be expected of medical colleges, even in the Confederacy, till the excitement of war abates. It is to be hoped that none of our medical colleges will be forced, even temporarily, to suspend. Medical men, well versed in the medical sciences, are important to the safety and comfort of the army, and, therefore, medical training is as important to the successful operation of our armies, in a protracted war, as military instruction itself. This fact will be appreciated when it is remembered that statistics of military campaigns prove that more soldiers perish from disease than from the sword.

We have not received circulars from all the Medical Colleges in the Confederate States, but hope it is not from any intention to suspend the next course. We have requested, by letter, the Dean of the Medical College of Georgia, at Augusta, to send us the Announcement of that institution for the next course of lectures, for the students here, and, having received none, fear none has been published. The organ of that institution, the "Southern Medical and Surgical Journal," has also failed to reach us for May, but as it has failed to appear once before during this year, from oversight, we hope nothing more has been the cause of the present failure. Cheer up, neighbors, our work is in support of the great cause of Southern Independence.

